# Superolateral capsule pathway: a new arthroscopic viewing approach for spotting femoral fixation device in anterior cruciate ligament reconstruction

**DOI:** 10.1186/s12891-024-08232-0

**Published:** 2024-12-27

**Authors:** Ming Ding, BingHui Liao, Lei Shangguan, YingChun Wang, Hu Xu

**Affiliations:** Sports Medicine Institution of Orthopaedics, The First Affiliated Hospital of Air Force Military Medical University, Xi’an, 710000 China

**Keywords:** Anterior cruciate ligament, Anterior cruciate ligament reconstruction, Knee arthroscopy, Pathway

## Abstract

**Background:**

This study aimed to describe the arthroscopic superlateral capsule pathway technique for spotting femoral fixation device deployment, and to compare the results with normal procedure.

**Methods:**

A total of 69 patients underwent ACLR (Anterior Cruciate Ligament Reconstruction) with or without the SCP (superolateral capsule pathway) during procedure were retrospectively selected and evaluated. A total of 36 patients underwent SCP and 33 patients underwent ACLR without SCP. Mean follow-up was 6 months after surgery. All patient noted joint fluid, underwent VAS and Lysholm score at follow-up, and statistical analysis was performed.

**Results:**

No statistically significant differences were found in patient demographics, ACLR duration time (*p* = 0.076) and Lysholm score (*p* = 0.296). Significantly less postoperation pain was reported in the SCP group (*p* = 0.000), and fluid volume in SCP group was significantly lower (*p* = 0.001). The postoperative complications were rare in both group.

**Conclusions:**

The superolateral capsule pathway approach is a minimally invasive and safe technique that can be used to accurately locate and implant suture button-based femoral fixation devices in anterior cruciate ligament reconstruction.

## Introduction

Anterior cruciate ligament (ACL) injury is a serious concern for physically active children and adolescents involved in sports, the incidence of which is increasing [1, 2]. According to the literature, the outcomes of anterior cruciate ligament reconstruction (ACLR) are better than those of nonsurgical treatment in terms of quality of life and function in sports [3, 4].

However, recent findings of a systematic review indicated that the ACLR failure rate is 0.72–1.76% [5]. Furthermore, the causes for ACLR failure were analyzed, and technical errors were found to be the most frequent cause [6].

Although suture button-based femoral cortical suspensory fixation can be used to provide correct graft fixation, several suture buttons failing to flip during the procedure have been reported [7, 8], which can compromise proper graft tension. Additionally, soft tissue interposition between the button and bone is associated with graft migration and pain, occasionally leading to failure and ACL revision [9]. Therefore, the button deployed on the lateral femoral cortex must be properly visualized by pulling on the trailing sutures. This technique is an arthroscopic technique to identify the proper deployment of suture button-based femoral fixation devices in anterior cruciate ligament reconstruction. The authors hypothesized that this technique might reduce surgical technical error and without effect on postoperative pain and knee function.

## Materials and methods

A retrospective study was conducted on patients who underwent arthroscopic ACLR between June 2022 and December 2022. All the procedures were performed by the same experienced surgeon.

Patients eligible for the study included those who underwent ACLR with or without the superolateral capsule pathway during procedure. The indication was ACL tear with or without meniscus tear; those with an ACL tear combined other ligament injury or associated fractures were excluded.

The patient demographics are reported in Table 1.

**Table 1:** Patient demographics

Demographics data of the study population	SCPG	NSCPG	Total	*p* value
Patients (no.)(%)	36(52.2)	33(47.8)	69	
Age(years)	29.63(± 7.77/20–50)	29.09(± 7.43/19–52)	29.37(± 7.56/19–52)	0.766
meniscus tear (with/without)(%)	31/5(86.11/13.89)	24/9(72.73/27.27)	55/14(79.71/20.29)	0.172

*Legend*: *No*. Number, *SCPG* Superolateral capsule pathway group, *NSCPG* None superolateral capsule pathway group

### Surgical technique

The patient was placed in a supine position under general or spinal anesthesia. Arthroscopic examination was performed through a standard anterolateral and anteromedial portal using a 30° arthroscope((Smith & Nephew, Oklahoma, USA). Pathological findings, including anterior cruciate ligament rupture and meniscus tear, were confirmed. After the diagnostic arthroscopy procedure, the ACL femoral footprint was identified and prepared with the preservation of the ACL remnant. Femoral and tibial tunnels were drilled using the anteromedial technique. Hamstring autografts were harvested using a tendon stripper and tubularized with No. 2-0 FiberWire. Then, the GraftLink was prepared using a suture button.

**Fig. 1:**
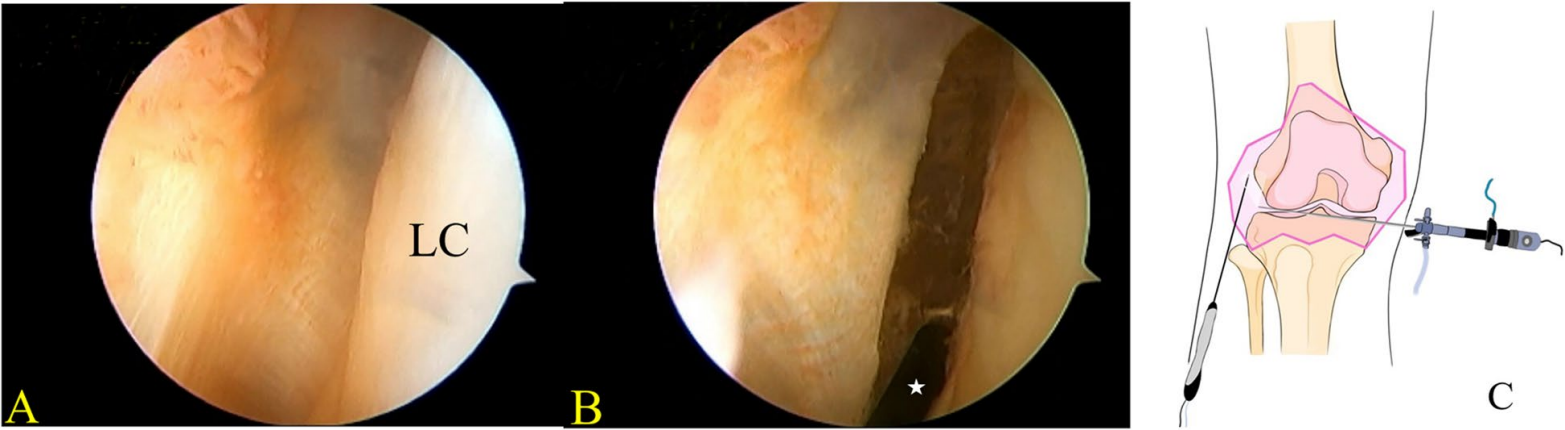
Make a pathway at superolateral capsule. **A** Viewing from the standard anteromedial portal. LC is femur lateral condyle. **B** Using radiofrequency probe (white star) to create a pathway at superolateral capsule through standard anterolateral portal. **C** Arthroscopic procedure sketch

**Fig. 2:**
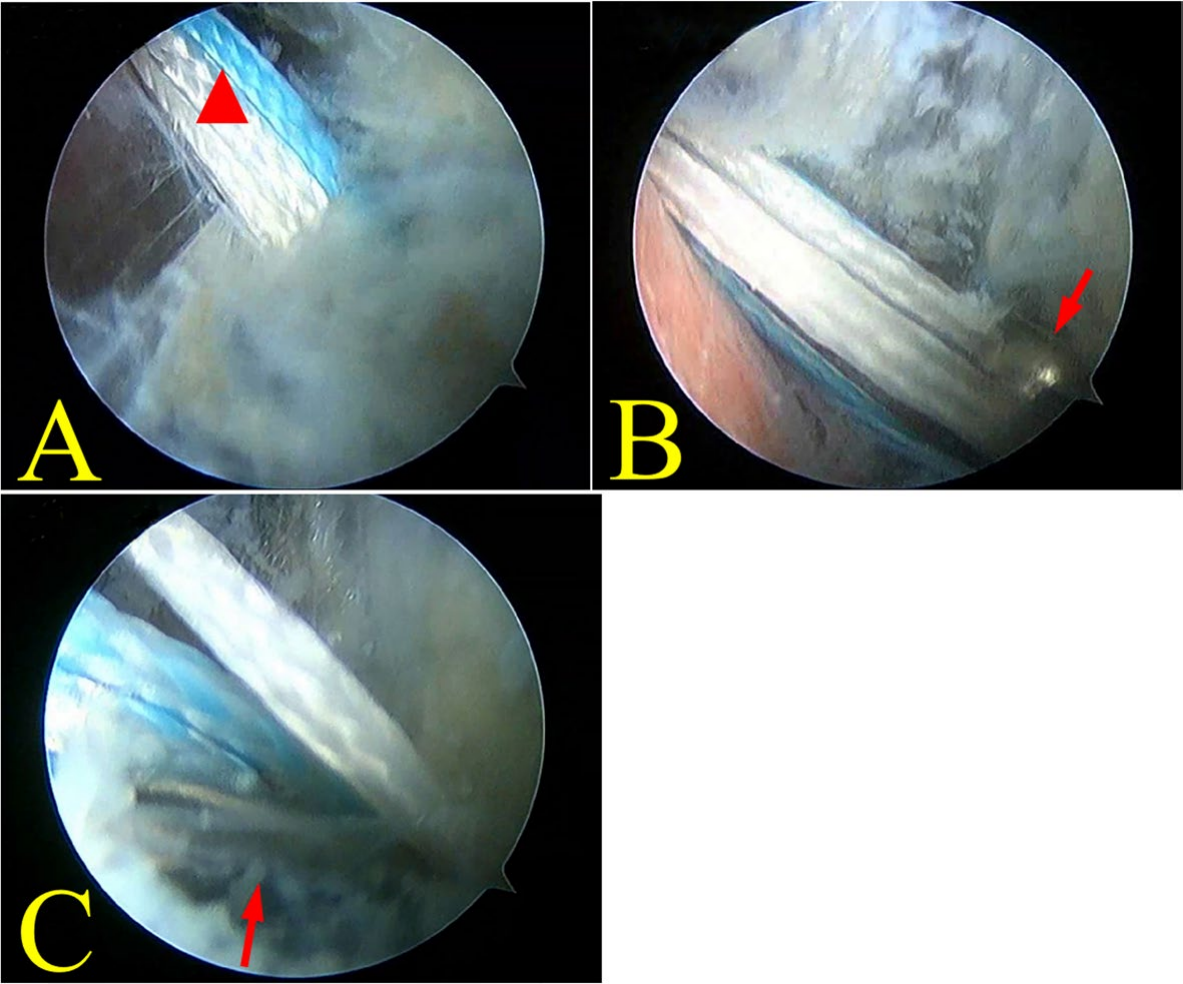
Flip suture button by viewing from superolateral capsule pathway. **A** The white traction sutures(triangle) allow to be visualized by viewing from superolateral capsule. **B** Button (arrow) could be visualized out the lateral femoral cortex. **C** Button (arrow) is spot to flip by pulling the flip suture

**Fig. 3:**
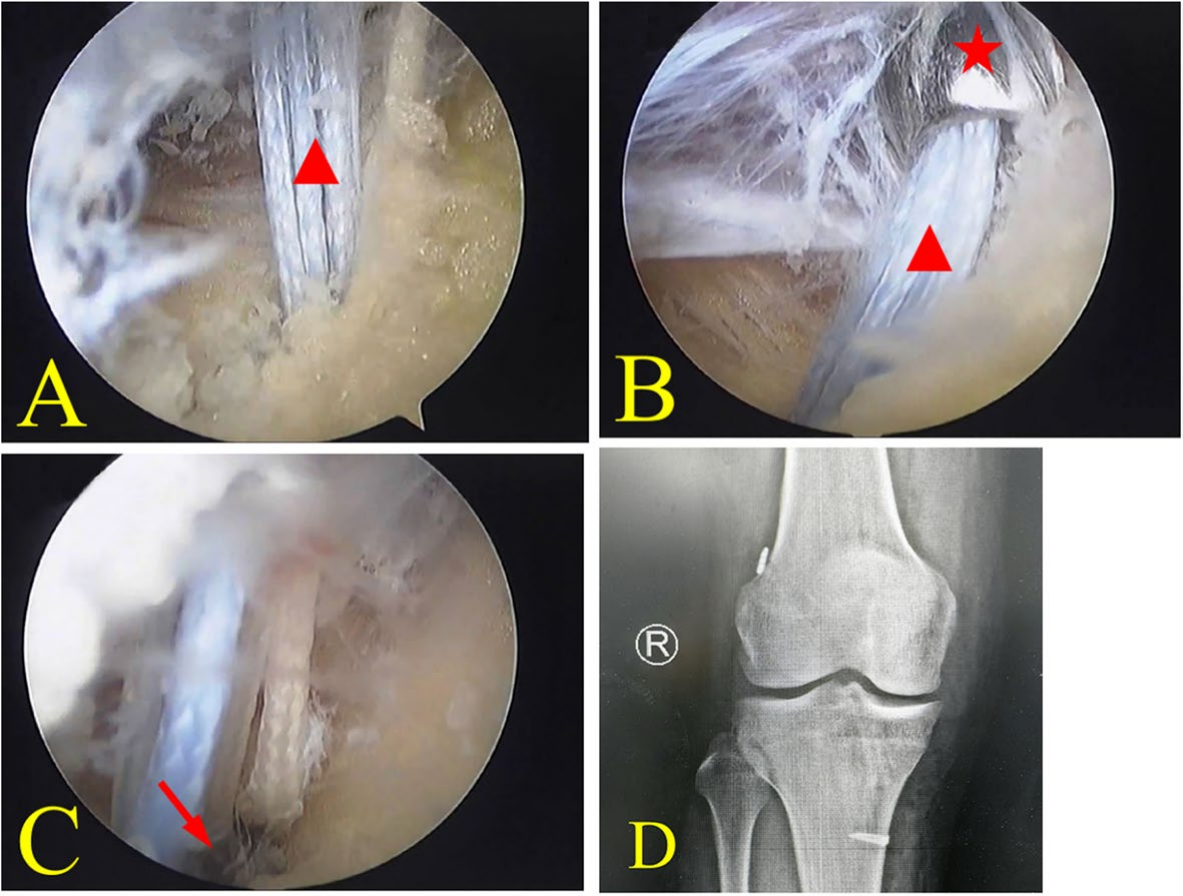
Readjust the overpulled adjustable loop suspension devices by viewing from superolateral capsule pathway. **A** Suspension devices is overpulled (triangle is the adjustable loop suture). In this situation, the button might get stuck by soft tissue. **B** Make a small skin incision to ensure arthroscopy sheath through outside-in along traction suture(star represents the arthroscopy sheath and triangle represents the adjustable loop suture). **C** Adjustable loop suspension devices is readjusted and successfully implant on the lateral femoral cortex(arrow is the button). **D** Post-operation X ray imagine

With the knee being left at approximately 30° of flexion, arthroscope was placed through the anteromedial portal to view the lateral gutter. A radiofrequency probe was used to form a pathway at the superolateral capsule (Fig. 1). Next, a switching stick was inserted to separate soft tissue, and a radiofrequency probe was used to coagulate blood. Then, the arthroscope was switched to the superolateral capsule pathway from the anterolateral portal using a switching stick. By viewing from the superolateral capsule, the traction sutures were visualized, and suture buttons were spotted to flip and implant on the lateral femoral cortex (Fig. 2). The superolateral capsule pathway enabled us to monitor the readjustment process of the adjustable loop suspension devices in instances of over-pulling. In such cases, the button could become lodged by soft tissue. To address this, a small skin incision is made to facilitate the passage of the arthroscopy sheath, providing an external-to-internal arthroscopic view along the traction suture. Subsequently, the graft is handled and the adjustable loop is tightened, allowing the button to be successfully implanted on the lateral femoral cortex (Fig. 3).

After the surgery, an indwelling drainage and a pressure bandage were applied to the knee joint. The drainage tube was removed on the second day after the operation, and swelling around the knee joint and the incision exudation were observed while performing quadriceps muscle strength exercise.

### Follow-up and function evaluation

All patients underwent postoperative pain assessment the day after surgery using a visual analogue scale (VAS) and noted joint fluid volume. Functional evaluation was performed using the Lysholm score at 6 months after surgery.

### Statistical analysis

Statistical analysis was performed using IBM SSPS statistics software version 22 (IBM, Armonk, NY, USA). An independent *t*-test was used to compare the patients’ demographics, surgery duration, VAS, fluid volume, and Lysholm score between SCPG and NSCPG. A *p* value < 0.05 was considered significant.

## Results

Between June 2022 and December 2022, a total of 69 patients (38 men and 31 women) were selected for this study. Patient age ranged from 19 to 52 years (mean 29.37 years). A total of 36 patients underwent SCP during the ACLR procedure: 20 men and 16 women, with age ranging from 20 to 50 years (mean, 29.63 years), of which 31 patients also had a meniscus tear. In total, 33 patients underwent NSCP during procedure: 18 men and 15 women, with age ranging from 19 to 52 years (mean, 29.09 years), of which 24 patients also had a meniscus tear. No statistically significant differences were found in patient demographics.

The mean VAS for those in the SCPG group was 1.167 (SD ± 0.378), whereas that for those in the NSCPG group was 1.969 (SD ± 0.809), indicating significantly less postoperation pain in the SCP group (*p* = 0.000). The mean fluid volume in the SCPG group was 56.944 (SD ± 20.677) mL, whereas that in the NSCPG group was 73.636 (SD ± 17.282) mL. The fluid volume after ACLR in the SCP group was significantly lower (*p* = 0.001). The mean Lysholm score was 86.555 (SD ± 2.347) in the SCP group and 87.151 (SD ± 2.346) in the NSCP group. The mean ACLR duration was 91.889 (SD ± 37.479) min in the SCP group and 79.242 (SD ± 15.518) min in the NSCP group.

A total of 12 patients reported postoperative complications: 4 in the SCP group (11.11%) and 8 in the NSCP group (24.24%). In the SCP group, one patient reported knee swelling (2.78%), and one patient reported knee stiffness (2.78%). Two patients were experiencing muscular atrophy at the 6-month follow-up but without functional deficit (5.56%). In those in the NSCP group, the most frequent complication was muscular atrophy (15.15%), followed by joint swelling (9.09%). All these patients demonstrated satisfactory function at the 6-month follow-up.

The clinical and functional results are reported in Table 2; the postoperative complications are reported in Table 3.

**Table 2:** Clinical and functional results of SCPG and NSCPG

	SCPG	NSCPG	*p* value
VAS	1.167 ± 0.378	1.969 ± 0.809	0.000
fluid volume(ml)	56.944 ± 20.677	73.636 ± 17.282	0.001
Lysholm score	86.555 ± 2.347	87.151 ± 2.346	0.296
surgery duration(min)	91.889 ± 37.479	79.242 ± 15.518	0.076

*Legend*: *VAS* The Visual Analogue Scale, *ml* milliliter, *min* minutes

**Table 3:** Postoperative complications

	SCPG	NSCPG
Complications	4(11.11%)	8(24.24%)
Inconstant pain	0	0
Swelling	1(2.78%)	3(9.09%)
Stiffness	1(2.78%)	0
Muscular atrophy	2(5.56%)	5(15.15%)
Arterial injury	0	0
Neurologic damage	0	0
Second surgical procedure	0	0

## Discussion

Currently, studies are reporting a high incidence of improper suture button deployment in ACLR. The postoperative rate of tissue interposition between the suture button and bone cortex reportedly ranges from 15% to 25.2% [7, 8]. An additional 9.8% of patients experience improperly flipped suture buttons during the ACLR procedure. Thus, in total, 25% of patients have improper suture button positioning without additional evaluation [9]. Conventionally, X-ray examinations are performed during surgery to ensure the correct placement of the suture button implant; however, this process prolongs the operation time and increases the risk of infection.Our study delineates the deployment and readjustment of a suture button via the superolateral capsule pathway. Prior to initiating this research, we conducted a review of previously published studies on similar topics [10–15]. Sonnery-Cottet B and Skelley N W, among others, have previously described the visualization and adjustment of the button within the lateral gutter [10, 11]. However, it is commonly understood that in most cases, the button cannot be directly visualized from the lateral gutter, as the lateral exit of the femoral tunnel is often situated in a proximal region, outside the capsule's attachment to the lateral femoral cortex. Previous studies have not established a superolateral capsule pathway; instead, they have suggested shaving soft tissue from the femoral tunnel outlet [10] or excising soft tissue between the button and the lateral femoral cortex using sharp scissors [12]. During our surgical procedure, we discovered that the superolateral capsule pathway offers clear visualization and ample space, enabling the safe and convenient readjustment of button placement. Additionally, some prior studies have outlined the alteration of button placement using a probe [10] or the tip of a guide pin [13]. In contrast, our study presents a method for readjusting an over-pulled button placement using an arthroscopic sheath, which provides sufficient force to reposition the button and guide it to the correct location through the sheath's canal. Our arthroscopic technique with a superolateral capsule pathway allows the clear visualization of the suture button flip and of the deployment on the lateral femoral cortex; previously, this procedure relied on surgeon experience. Our recommended technique enhances the success rate of suture button deployment. The indications for this technique are as follows: (1) observation of suture button flip during ACL reconstruction, and (2) observation of overpulled suture button adjustment to correct the location on the lateral femoral cortex. The advantages and disadvantages of the technique are presented in Table 4. Procedural benefits and limitations are listed in Table 5.

**Table 4:** Advantages and disadvantages of superolateral capsule pathway

**Advantages**
This minimally pathway can facilitate faster recovery
Direct visualization of suture button-based femoral fixation device implant in anterior cruciate ligament reconstruction
Direct visualization of over-pulled suture button adjust to correct lateral femoral cortex place
**Disadvantages**
This pathway may increase the risk of fluid extravasation
Radiofrequency probe may increase the risk of skin and subcutaneous tissure burn injuries

**Table 5:** Procedural benefits and limitations

**Pearls**
The pathway position can be adjusted in the anterior–posterior lateral sulcus area according to the femoral tunnel technique (Anterior medial technique can adjusted in the posterior for the posterior tunnel position)
Use switching stick separate tissue space to avoid damaging the traction line
**Pitfalls**
Potential risk of lateral superior genicular artery injury
Carefully removing the soft tissue from the lateral aspect of the femoral cortex

ACL rupture is a debilitating injury, and the subsequent ACLR results in the passive stability of the knee, leading to good patient-reported outcomes in the short-to-medium term [16]. However, even with appropriate surgery and rehabilitation, only approximately 65–75% of recreational ACLR patients return to their preinjury sporting level [17]. Quadriceps atrophy and weakness of the surgical leg are considered to cause side-to-side strength asymmetry and poor rehabilitation, affected by postoperative pain [18, 19]. Buckthorpe et al. [19, 20] suggested a numeric rating scale pain value of 0–2 (knee specific) as a criterion for transition to higher-intensity rehabilitation. In our study, postoperative fluid volume and VAS were significantly lower in those in the SCP group, possibly because the postoperative intra-articular bleeding was drained to the deep surface of the iliac tendon bundle through the SCP incision. This technique allows intra-articular drainage, effectively alleviates postoperative swelling, and reduces synovial pain in the surgical knee, effectively reducing functional interference to the quadriceps and enhancing early recovery of knee function.

The incidence of complications in the SCP group was 11.11%, which is significantly lower than that in the NSCP group. The most common symptom in the SCP group was quadriceps atrophy (5.56%), followed by joint swelling (2.78%) and joint stiffness (2.78%), but the incidence of these symptoms was significantly lower than that in the NSCP group. Other complications may include lateral fluid exosmosis and retention because this technique is performed in the extraarticular space. Additionally, damage to the button traction sutures, caused by the inappropriate use of a radiofrequency probe, may occur.

## Conclusions

The superolateral capsule pathway technique was developed to visualize flip and implant suture buttons directly on the lateral femoral cortex under arthroscopy . This technique offers lower postoperation pain and joint fluid volume with insignificant ACLR duration and postoperation function differences. The superolateral capsule pathway approach is a minimally invasive and safe technique may help avoid the failure of suture button-based femoral fixation devices deployment in anterior cruciate ligament reconstruction.

## Data Availability

No datasets were generated or analysed during the current study.
